# Safety and immunogenicity of diphtheria, tetanus and pertussis (acellular, three components) combined vaccine, adsorbed after three-dose priming in 2 months age infants: a randomized, blinded, controlled phase III clinical trial in China

**DOI:** 10.1080/22221751.2026.2625556

**Published:** 2026-01-30

**Authors:** Wei Zhang, Chen Wei, Peng Wan, Guangwei Feng, Feiyu Wang, Lichan Wang, Jiebing Tan, Xuewen Wang, Xue Wang, Xiuwen Sui, Wangyang You, Jinbo Gou, Liyong Yuan, Tao Zhu, Haitao Huang, Xiao Ma, Yanxia Wang

**Affiliations:** a Henan Province Center for Disease Control and Prevention, Zhengzhou, People’s Republic of China; b National Institutes for Food and Drug Control, Beijing, People’s Republic of China; c CanSino Biologics Inc., Tianjin, People’s Republic of China; d Shanghai Imstat medical technology CO., LTD, Shanghai, People’s Republic of China

**Keywords:** Diphtheria, tetanus and pertussis (acellular, three components) combined vaccine, adsorbed, infant immunization, safety, immunogenicity, clinical trial

## Abstract

Pertussis remains a leading cause of infant mortality globally, with rising cases in China post-COVID-19. Despite vaccination, waning immunity from acellular pertussis vaccines has driven resurgence. We evaluated a novel diphtheria, tetanus and pertussis (acellular, three components) combined vaccine, adsorbed (DTcP), China’s first genetically engineered three-component pertussis vaccine, administered under two primary schedules (2/3/4 vs. 2/4/6 months) compared to licensed DTaP-IPV-Hib (Pentaxim). In this randomized, blinded, phase III clinical trial, 1380 healthy 2-month-old infants from Henan, China, received DTcP-1 (2/3/4 months) or DTcP-2 (2/4/6 months) or DTaP-IPV-Hib (2/3/4 months). The primary safety endpoints were the incidence of adverse reactions within 0∼30 days after primary vaccination. The primary immunogenicity endpoints were to evaluate the non-inferiority and superiority of seroconversion rates and geometric mean concentrations (GMCs) of anti-pertussis toxoid (PT), filamentous hemagglutinin (FHA), pertactin (PRN), diphtheria toxoid (DT), tetanus toxoid (TT), antibodies 30 days after primary vaccination. Immunogenicity was assessed via Luminex-based multiplex immunoassay. Both DTcP-1 and DTcP-2 schedules demonstrated non-inferior safety to DTaP-IPV-Hib (total adverse reactions: 14.52/16.59% vs. 16.91%, *P* = 0.183), with DTcP-2 (2/4/6 months) showing lower swelling (2.59% vs. 4.83%, *P* = 0.008) and irritability (0.07% vs. 1.02/1.40%, *P* < 0.001). DTcP-2 elicited higher GMCs against pertussis antigens (PT: 84.23 vs. 65.35; FHA: 132.16 vs. 102.13, both *P* < 0.001) and comparable DT/TT responses. DTcP exhibited favourable safety and superior pertussis immunogenicity, particularly with the 2/4/6-month schedule. Its genetically engineered three-component design offers a promising strategy to combat pertussis amid global resurgence.

## Introduction

Pertussis (whooping cough), diphtheria, and tetanus are vaccine-preventable infectious diseases that continue to pose significant public health challenges, particularly among infants and young children in regions with inadequate immunization coverage or in populations with waning vaccine-induced immunity [1]. In China, pertussis has re-emerged as a significant public health concern, showing a notable resurgence in the population after the COVID-19 pandemic, following the official end of China's zero-COVID-19 policy on 8 January 2023 [2]. Reported cases were 30,027 in 2019 and remained stable in 2020–2021 [3]. However, surveillance data revealed a sharp rise to 476,690 cases in 2024 (more than 12-fold higher than 38,205 of 2023), including 31 deaths [4–7]. Besides, global data indicate that the majority of pertussis-related deaths occur in infants younger than 3 months. For example, from January to 10 May 2024, a pertussis outbreak in Italy resulted in 108 hospitalizations and three deaths, according to a report from 11 pediatric hospitals. The most affected group was unvaccinated infants and newborns [8]. These findings underscore the critical need for targeted strategies to protect infants, who remain the most at-risk population for severe pertussis complications [9]. A recent increase in pertussis cases among older children, several years after they have completed their primary vaccination series, has been observed in nations using purified acellular pertussis vaccines. This resurgence has been attributed to inadequate or declining immunity provided by these purified acellular pertussis vaccines [10]. Moreover, previous study compared the positive rate of acellular pertussis vaccines between co-purified and component diphtheria-tetanus-pertussis (DTP) vaccines. It was indicated that the positive rate of component DTP vaccine reached 84.44%, which was significantly higher than that of co-purified DTP vaccine (37.22%) [11].

Diphtheria and tetanus remain significant threats to infant health globally, despite being vaccine-preventable diseases. The disease burden remains disproportionately high in regions with suboptimal vaccination coverage, where infant immunization programmes face implementation challenges. Fortunately, the general population is susceptible to diphtheria, and infection confers long-lasting immunity. Since China implemented routine immunization in 1978, diphtheria incidence has declined annually. From 2017 to 2023, there were 93–19 cases of neonatal tetanus, with one fatality. In 2024, there were 14 cases [12].

Previously, the primary vaccination of DTP was administered at 3, 4 and 5 months, with one-month interval. As of 1 January 2025, the immunization schedule for the national immunization programme's DTP vaccine has been adjusted to a 3-dose prim regimen, with doses administered at 2, 4, and 6 months [13]. Given the ongoing burden of pertussis, diphtheria, and tetanus in infants, there is a critical need for safe and effective vaccines that can be administered early in life, thereby the vaccination programmed was changed to start at 2-month-old. This study evaluated the safety and immunogenicity of a novel diphtheria, tetanus and pertussis (acellular, three components) combined vaccine, adsorbed (DTcP) in 2-month-old infants, comparing it to the currently used DTaP-IPV-Hib vaccine. Notably, this DTcP vaccine, produced through a gene expression-based purification and detoxification process, represents a domestically developed product to complete standardized clinical trials using (2, 4, 6 months) primary immunization schedules. The safety and immunogenicity outcomes were systematically analysed across these different vaccination schedules to provide comprehensive evidence for immunization strategy optimization.

## Methods

### Study design

This is a randomized, blinded, and controlled trial, sited in six cities/counties of Henan province, China from 11st August 2023 to 31st July 2024. The study recruited 2-month-old (60–89d) infants who have not been vaccinated with pertussis, diphtheria, tetanus, IPV, Hib, or 13-valent pneumococcal polysaccharide conjugate vaccine. The exclusion criteria included infants who are born prematurely (delivered before the 37th week of pregnancy), with low birth weight (birth weight < 2500 g); with abnormal labour, history of asphyxia rescue, and history of nervous system damage; who have suffered from one of the diseases of pertussis, diphtheria or tetanus; who have had contact with individuals diagnosed with pertussis, diphtheria, or tetanus in their families in the past 30 days; have a history of allergy to any of the vaccines or vaccine components, and have serious side effects to vaccines, such as allergies, urticaria, dyspnea, angioedema; with a history of epilepsy, convulsions, cerebral palsy, or a history of mental illness or family history; or other progressive neurological diseases; has been diagnosed with congenital or acquired immunodeficiency, HIV infection, lymphoma, leukemia, systemic lupus erythematosus (SLE), juvenile rheumatoid arthritis (JRA), or other autoimmune disease; asplenia caused by any condition, defects in spleen function; known or suspected acute illness or severe chronic disease (including: severe respiratory disease, severe cardiovascular disease, liver and kidney disease, severe skin disease, malignant tumour, etc.); or in the acute phase of chronic disease; physician-diagnosed coagulation abnormalities (such as coagulation factor deficiencies, coagulation disorders, platelet abnormalities) or significant bruising or coagulation disorders; immunosuppressants or modulators, cytotoxic continuous treatment for more than 10 days (except for inhaled and topical steroids) before receiving the investigational vaccine; received blood products (other than hepatitis B immune globulin) prior to receiving the investigational vaccine; receipt of other investigational drugs or investigational vaccines within 1 month prior to receiving the investigational vaccine; plan to participate in or be participating in any other drug clinical study; received a live attenuated vaccine within 14 days before receiving the investigational vaccine, or other vaccines within 7 days; axillary body temperature > 37.0°C prior to vaccination; any other factors that are not suitable for participation in the clinical trial as judged by the investigator. For the second, third, and fourth doses, those who have had a severe allergic reaction after the previous dose of vaccination; with serious adverse reactions that have a causal relationship with the previous dose of vaccination; who are newly discovered or newly discovered after the first vaccination do not meet the inclusion criteria for the first dose or meet the exclusion criteria for the first dose shall be determined by the investigator whether to continue to participate in the study; other reasons for exclusion in the opinion of the investigator were excluded.

In this study, participants aged 2 months (60–89d) received three doses of primary vaccination, and one dose of booster vaccination at the age of 18–24 months. SAS 9.4 software was performed for random grouping. Randomization was performed at 2 months of age using block randomization with a block size of 6. Eligible participants were randomly assigned to DTcP-1 group (primary vaccination at 2, 3, and 4 months), DTaP-IPV-Hib group (primary vaccination at 2, 3, and 4 months,) and DTcP-2 group (primary vaccination at 2, 4, and 6 months) according to the ratio of 1:1:1. Participants were unblinded after 7 days of safety data collection. participants in DTcP-1 or DTcP-2 experimental groups would subsequently receive IPV and Hib vaccines according to the national immunization programme's vaccination strategy. Blood samples were collected before the first dose of vaccination, 30d after three doses of primary vaccination, before booster vaccination and 30d after booster vaccination. The protocol and informed consent were approved by the ethics committee of Henan Province Center for Disease Control and Prevention. Study was performed in accordance with the principles stated in the Declaration of Helsinki, and was registered on the ClinicalTrial.gov (NCT05951725). Written informed consent was obtained before enrolment. The primary immunogenicity endpoints were to evaluate the non-inferiority and superiority of seroconversion rates and geometric mean concentrations (GMCs) of anti-pertussis toxoid (PT), filamentous hemagglutinin (FHA), pertactin (PRN), diphtheria toxoid (DT), tetanus toxoid (TT), antibodies 30 days after primary vaccination. Geometric mean increases (GMIs) and seropositive rate of FHA, PRN, DT, TT antibodies 30 days after the primary vaccination were the secondary objectives. The primary safety endpoints were the incidence of adverse reactions within 0∼30 days after primary vaccination.

### Luminex

The antibody responses to pertussis (PT, FHA, and PRN), diphtheria, and tetanus antigens were quantitatively measured using a multiplexed fluorescent immunoassay based on Luminex technology. This high-throughput methodology employs antigen-conjugated magnetic microspheres, where each bead region is uniquely coded and coated with specific vaccine antigens. Reference sera were traceable to the 1st International Reference Preparation 06/140, and the national human antiserum reference material of China was used, with coated antigens sourced from the national reference antigens of China. During the assay, serum samples were incubated with the mixed antigen-coupled beads, allowing specific antibodies to bind to their respective antigens. Following washing steps to remove unbound components, phycoerythrin-conjugated detection antibodies were introduced to form fluorescent immune complexes. The Luminex analyzer simultaneously identified each microsphere region and quantified the bound antibodies. Standard curves were generated using an 8-point dilution series of reference sera, with data analysis performed five-parameter logistic regression. Each analytical run included one high-level and one low-level quality control serum, and the coefficient of variation for quality control sera was required to be less than 20%. All samples were tested at three optimized dilutions in duplicate, with geometric mean antibody concentrations calculated after excluding outliers demonstrating >20% coefficient of variation between replicates. Samples with antibody concentrations outside the quantitative range were re-tested at appropriate dilutions to ensure accurate measurement.

### Vaccines

The experimental vaccine DTcP were produced by Cansino Bio Inc, which was developed by modifying the existing pertussis vaccine production strain (CMCC58003) using genetic engineering techniques consistent with internationally marketed vaccines. Three genetically engineered pertussis strains expressing FHA, PT, and PRN antigens were constructed, followed by comprehensive clone screening with verification of both genotype and phenotype. The final vaccine formulation was prepared by mixing and diluting five bulk antigens (FHA, PT, PRN, DT and TT) in appropriate proportions. A novel dual-adjuvant adsorption process was employed, incorporating both aluminum hydroxide and aluminum phosphate adjuvants. The control vaccine DTaP-IPV-Hib was produced by Sanofi Pasteur, which containing four antigens (FHA, PT, DT and TT). The package contains two components, one is Diphtheria, Tetanus, Pertussis, and Poliomyelitis Vaccine, suspension, 0.5 ml per vial, the other is Haemophilus influenzae type b (Hib) Conjugate Vaccine, lyophilized powder. Before use, the two components must be mixed. Each dose was 0.5 ml and was taken intramuscularly. Among them, the vaccination site of the participants under 12 months of age was the anterolateral thigh, and the vaccination site of the participants aged 12 months and above was the deltoid muscle of the upper arm.

### Statistical analysis

Sample size calculation based on estimated antibody seroconversion rates was performed using the “Confidence Intervals for One Proportion” feature in NCSS-PASS (Version 16.0). To account for comparisons between different immunization regimens, the probability of Type I error was adjusted. α = 0.0125 (one-tailed), with a two-tailed 97.5% confidence interval lower limit not less than 80% of the lowest PT or FHA group in the control cohort. Assuming a post-vaccination PRN antibody seroconversion rate of no less than 80%, with an interval precision set at 0.1, the calculated sample size required is 340 cases. Accounting for a 20% dropout rate, each group requires 460 cases.

For safety statistical analysis, MedDRA was used for the medical coding of adverse events and serious adverse events, which were then classified and statistically analysed at the System Organ Class (SOC) and Preferred Term (PT) levels. The evaluation of the data primarily included summarizing clinical response endpoints and the incidence rates of local and systemic clinical reactions in all participants during the observation period. In terms of immunogenicity analysis, antibody concentrations were log-transformed, and one-way ANOVA was used to compare the GMCs of pre-vaccination antibodies across groups to determine differences. For non-inferiority comparisons, the two-sided 97.5% confidence interval (CI) for the difference in seroconversion rates of anti-PT and anti-FHA antibodies between the DTcP group and the DTaP-IPV-Hib control group was calculated, with non-inferiority established if the lower limit of the CI was greater than – 0.10, and superiority was determined if the lower limit of the two-sided 97.5% CI exceeded 0. Additionally, the two-sided 97.5% CI for the post-vaccination seroconversion rate of anti-PRN antibodies in the experimental group was calculated, and if this rate was not less than 80% of the lower seroconversion rate among the PT and FHA components in the control vaccine, the anti-PRN antibody was considered to have acceptable immunogenicity. The chi-square test, corrected chi-square test, or Fisher's exact test was used to compare the seroconversion rates of anti-DT and anti-TT antibodies between the experimental and control groups, with non-inferiority established if the lower limit of the experimental group's two-sided 97.5% CI was not less than 80% and 85%. Furthermore, the two-sided 97.5% CI for the ratio of antibody GMCs between the DTcP group and the DTaP-IPV-Hib control group was calculated, with non-inferiority established if the lower limit of the CI for the GMC ratio was ≥0.67, and superiority was determined if the lower limit of the two-sided 97.5% CI exceeded 1. These analyses were performed separately for the DTcP (2, 4, 6-month schedule) compared to the control group and for the DTcP (2, 3, 4-month schedule) compared to the control group, with non-inferiority testing conducted for both comparisons. Descriptive and statistical comparisons were additionally made for the seroconversion rates and GMCs of anti-PT, anti-FHA, anti-DT, anti-TT, and anti-PRN antibodies between the experimental group (2, 4, 6-month schedule) and the experimental group (2, 3, 4-month schedule). According to the definition of seroconversion, for anti-DT and TT antibodies, participants with pre-vaccination antibody concentrations <0.1 IU/ml, post-vaccination concentrations ≥0.1 IU/ml are considered positive. For participants with pre-vaccination concentrations ≥0.1 IU/ml, a fourfold increase in post-vaccination concentration is regarded as seroconversion. For anti-PT, FHA, and PRN antibodies, for pre-vaccination antibody concentrations <5 IU/ml, post-vaccination concentrations ≥20 IU/ml; for pre-vaccination concentrations ≥5 IU/ml but <20 IU/ml, a fourfold increase in post-vaccination concentration; for pre-vaccination concentrations ≥20 IU/ml, a fourfold increase in post-vaccination concentration is considered seroconversion. The criteria for antibody positivity were: Anti-PT and FHA antibodies ≥20 IU/ml; anti-DT and TT antibodies ≥0.01 IU/ml. As no established positive standard exists for anti-PRN antibodies, this study provisionally sets the positive threshold at ≥5 IU/ml.

## Results

**Figure 1.**
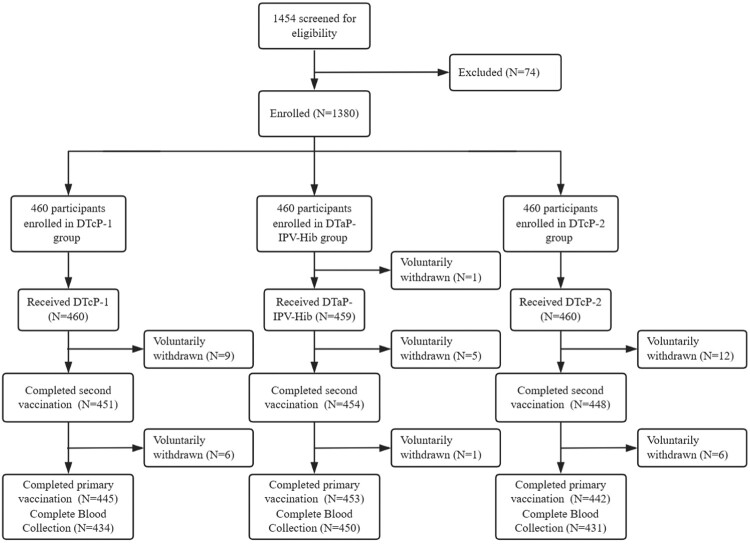
Flow diagram.

In this study, a total of 1454 participants were screened, with 1380 participants were enrolled. Ultimately, 1379 subjects were completed first dose, and 1340 individuals completed primary vaccination (Figure 1). The demographic characteristics of the three study groups were well-balanced and comparable (Table 1). The mean ages in the DTcP-1 group, DTaP-IPV-Hib group and DTcP-2 group were 2.42, 2.42 and 2.40 months, respectively. In the DTcP-1 group, the mean height and weight were 60.60 cm and 6.28 kg respectively, and 5.43% reported previous medical history. In the DTaP-IPV-Hib group, the mean height was 60.64 cm and weight was 6.32 kg, and the prevalence of medical history was 5.66%. The DTcP-2 group demonstrated comparable demographics, with a mean height of 60.68 cm, mean weight of 6.27 kg, and the prevalence of previous medical history of 4.35%. Overall, the groups were well-balanced in terms of age, gender distribution, height, weight, and medical history prevalence.

**Table 1.** Demographic characteristics.

	DTcP-1 (n = 460)	DTaP-IPV-Hib (n = 459)	DTcP-2 (n = 460)
Age (month)	2.42(0.26)	2.42(0.27)	2.40(0.26)
Gender n (%)			
Female	237(51.52)	228(49.67)	222(48.26)
Male	223(48.48)	231(50.33)	238(51.74)
Height (cm)	60.60(2.35)	60.64(2.31)	60.68(2.43)
Weight (kg)	6.28(0.75)	6.32(0.75)	6.27(0.74)
Previous medical history n (%)			
Yes	25(5.43)	26(5.66)	20(4.35)
No	435(94.57)	433(94.34)	440(95.65)

Note: n represents the number of participants received first dose vaccine in each group. The numbers in parentheses were the SD value.

### Safety

Overall, the proportion of participants experiencing any adverse reaction was similar among the groups, with rates of 16.59% in DTcP-1, 16.91% in DTaP-IPV-Hib, and 14.52% in DTcP-2 (*P* = 0.183) (Table 2). Importantly, the majority of these reactions (74.4%−76.3%) occurred within the first 3 days post-vaccination, with only 3.73%−4.22% occurring between days 4–7 (Table 3). Grade 3 or higher adverse reactions were rare, occurring in 0.66%, 0.66%, and 0.59% of participants, respectively (*P* = 0.967), and were similarly concentrated in the first week.

**Table 2.** Overview of adverse reactions within 30 days after primary vaccination (SS).

	DTcP-1 (N = 1356)	DTaP-IPV-Hib (N = 1366)	DTcP – 2 (N = 1350)	*P*
All adverse reactions	225(16.59%)	231(16.91%)	196(14.52%)	0.183
Grade 3	9(0.66%)	9(0.66%)	8(0.59%)	0.967
Local adverse reactions	82(6.05%)	82(6.00%)	57(4.22%)	0.057
Systemic adverse reactions	151(11.14%)	154(11.27%)	142(10.52%)	0.799
Swelling	57(4.20%)	66(4.83%)	35(2.59%)	**0**.**008**
Grade 3	0	0	1(0.07%)	0.332
Erythema	43(3.17%)	34(2.49%)	33(2.44%)	0.425
Grade 3	0	0	1(0.07%)	0.332
Induration	6(0.44%)	8(0.59%)	4(0.30%)	0.524
Fever	45(3.32%)	65(4.76%)	70(5.19%)	**0**.**047**
Grade 3	2(0.15%)	0	2(0.15%)	0.406
Irritability	19(1.40%)	14(1.02%)	1(0.07)	**<0**.**001**
Diarrhea	32(2.36%)	22(1.61%)	25(1.85%)	0.351
Grade 3	2(0.15%)	0	1(0.07)	0.331
Vomit	9(0.66%)	9(0.66%)	8(0.59%)	0.967
Nausea	0	0	1(0.07%)	0.332
Cough	53(3.91%)	56(4.10%)	60(4.44%)	0.778
Grade 3	5(0.37%)	9(0.66%)	4(0.30%)	0.320
Drowsiness	5(0.37%)	6(0.44%)	1(0.07%)	0.177

Note: *P*-values were derived from χ2 test or Fisher's exact test for categorical variable comparisons between groups. N represents the total number of participants per dose across all groups.

**Table 3.** Duration of adverse reactions 0–30 days after vaccination.

	DTcP-1 (N = 1356)	DTaP-IPV-Hib (N = 1366)	DTcP-2 (N = 1350)	*P*
**Total**	225(16.59%)	231(16.91%)	196(14.52%)	0.183
1-3d	166(12.24%)	172(12.59%)	137(10.15%)	0.101
4-7d	52(3.83%)	51(3.73%)	57(4.22%)	0.788
**Solicited**	223(16.45%)	227(16.62%)	190(14.07%)	0.127
1-3d	166(12.24%)	170(12.45%)	135(10.00%)	0.087
4-7d	50(3.69%)	50(3.66%)	54(4.00%)	0.876
**Local**	82(6.05%)	82(6.00%)	57(4.22%)	0.057
1-3d	62(4.57%)	61(4.47%)	41(3.04%)	0.076
4-7d	16(1.18%)	22(1.61%)	19(1.41%)	0.633
**Systemic**	151(11.14%)	154(11.27%)	142(10.52%)	0.799
1-3d	107(7.89%)	114(8.35%)	100(7.41%)	0.663
4-7d	35(2.58%)	28(2.05%)	37(2.74%)	0.476

Note: N represents the total number of participants per dose across all groups.

Local adverse reactions were reported in 6.05% (DTcP-1), 6.00% (DTaP-IPV-Hib), and 4.22% (DTcP-2) of participants (*P* = 0.057), with 75.5%−80.3% emerging within 1–3 days. Swelling was significantly more frequent in the DTaP-IPV-Hib group (4.83%) compared to DTcP-2 (2.59%, *P* = 0.008), while erythema and induration showed no significant differences. Systemic adverse reactions occurred in 11.14% (DTcP-1), 11.27% (DTaP-IPV-Hib), and 10.52% (DTcP-2) of participants (*P* = 0.799), predominantly (70.8%−74.1%) within the first 3 days. The most common systemic adverse reactions were fever, diarrhea and cough. Among them, there was a significant difference in the incidence of fever among three groups, where participants in DTcP-2 group having the highest rate (5.19%) compared to DTcP-1 (3.32%) and DTaP-IPV-Hib (4.76%, *P* = 0.047). Irritability was significantly less frequent in DTcP-2 (0.07%) than in the other groups (*P* < 0.001). Other systemic reactions, including diarrhea, vomiting, cough, and drowsiness, showed no statistically significant differences.

The temporal pattern demonstrated that approximately 90% of all adverse reactions resolved within 7 days, with the most intense period being 1–3 days post-vaccination. On the whole, adverse reaction profiles were generally similar across the three vaccines, and most adverse reactions were transient in nature. The incidence of adverse reactions in all groups was low, and the grades of adverse reactions were mainly mild and moderate. The vast majority of adverse reactions were solicited, and no vaccine-related serious adverse events occurred.

### Immunogenicity

The seroconversion rates for anti-PT and anti-FHA antibodies were assessed for non-inferiority and superiority 30 days after primary vaccination (Table 4). For both DTcP-1 and DTcP-2 compared to the licensed DTaP-IPV-Hib vaccine, non-inferiority was conclusively demonstrated for both PT and FHA. All point estimates for the difference in seroconversion rates were positive (ranging from 1.02% to 2.66%), and the lower bounds of the 97.5% CIs (ranging from −2.32% to −0.26%) were well above the −10% non-inferiority margin. Consequently, the null hypothesis for non-inferiority was rejected in all four comparisons. Following the confirmation of non-inferiority, superiority was tested. In none of the comparisons was statistical superiority established, as the lower bounds of all 97.5% CIs included or fell below 0. Therefore, while the DTcP vaccines induced seroconversion rates that were not inferior to the comparator, they were not shown to be statistically superior.

**Table 4.** Non-inferiority/superiority comparison of seroconversion rates of anti-PT and FHA antibodies.

	Point Estimate (%)	97.5%CI	Non-inferiority Cut-off (%)	Established or not	Superiority Cut-off	Established or not
30 days after primary vaccination						
DTcP-1 VS DTaP-IPV-Hib						
PT	2.66	(−0.26,5.59)	−10	Yes	0	No
FHA	1.36	(−1.91,4.62)	−10	Yes	0	No
DTcP-2 VS DTaP-IPV-Hib						
PT	1.83	(−1.27,4.94)	−10	Yes	0	No
FHA	1.02	(−2.32,4.37)	−10	Yes	0	No

Note: When the lower bound of two-sided the 97.5% CI on both sides of the point estimate ≥ −0.1, the non-inferiority is established. The superiority is established when the lower bound of the two-sided 97.5% CI is >0. When the non-inferiority test is established, the superiority test is performed.

Table 5 illustrated the seroconversion rates of antibodies 30 days after primary vaccination for DTcP-1, DTaP-IPV-Hib, and DTcP-2. High seroconversion rates were observed across all groups for DT and TT, with rates exceeding 99% and no significant differences between DTaP-IPV-Hib and DTcP-1 (DT: 99.05% vs. 99.23%, *P* > 0.999; TT: 99.29% vs. 99.23%, *P* > 0.999) or between DTaP-IPV-Hib and DTcP-2 (DT: 99.05% vs. 99.74%, *P* = 0.432; TT: 99.29% vs. 100.00%, *P* = 0.284). For pertussis-related antibodies, seroconversion rates were also high but varied by component. Comparing DTaP-IPV-Hib and DTcP-1, the seroconversion rate for PT was significantly higher in DTcP-1 (97.69% vs. 95.02%, *P* = 0.045), while FHA rates were comparable (94.79% vs. 96.14%, *P* = 0.355). In contrast, comparisons between DTaP-IPV-Hib and DTcP-2 showed no significant differences for PT (95.02% vs. 96.86%, *P* = 0.190) or FHA (94.79% vs. 95.81%, *P* = 0.494). These results highlighted that while all vaccines achieved high seroconversion for DT and TT, and DTcP-1 additionally showed a modest but significant advantage over DTaP-IPV-Hib in PT seroconversion.

**Table 5.** Seroconversion rate of antibodies 30 days after primary vaccination.

	DTcP-1	DTaP-IPV-Hib	DTcP-2	P*	*P*#
DT	99.23% (97.76,99.84)	99.05% (97.59,99.74)	99.74% (98.55,99.99)	>0.999	0.432
TT	99.23% (97.76,99.84)	99.29% (97.94,99.85)	100.00% (99.04,100.00)	>0.999	0.284
PT	97.69% (95.65,98.94)	95.02% (92.49,96.89)	96.86% (94.58,98.37)	**0**.**045**	0.190
FHA	96.14% (93.72,97.83)	94.79% (92.21,96.70)	95.81% (93.29,97.59)	0.355	0.494
PRN†	98.71% (97.03,99.58)	0.95% (0.26,2.41)	99.48% (98.12,99.94)	**<0**.**001**	**<0**.**001**

* Comparison between DTaP-IPV-Hib and DTcP-1 groups.

# Comparison between DTaP-IPV-Hib and DTcP-2 groups.

† PRN antigen is a component of DTcP but is absent from the DTaP-IPV-Hib.

The numbers in parentheses were the 95% CI.

Table 6 demonstrated the GMCs of non-inferiority and superiority 30 days after primary vaccination for anti-PT and anti-FHA antibodies. For both DTcP-1 and DTcP-2, non-inferiority was established for both PT and FHA. The GMC ratios for PT were 1.16 [1.05, 1.27] and 1.29 [1.17, 1.42], and for FHA were 0.93 [0.84, 1.03] and 1.29 [1.17, 1.43]. However, anti-FHA antibodies did not meet the superiority criterion (point estimate = 0.93; 97.5% CI: 0.84-1.03). In the DTcP-2 versus DTaP-IPV-Hib comparison, both anti-PT (point estimate = 1.29; 97.5% CI: 1.17-1.42) and anti-FHA (point estimate = 1.29; 97.5% CI: 1.17-1.43) antibodies showed statistically significant superiority, with the lower bounds of their CIs exceeding 1.

**Table 6.** Non-inferiority/superiority comparison of GMCs of anti-PT and FHA antibodies.

	Point Estimate	97.5%CI	Non-inferiority Cut-off	Established or not	Superiority Cut-off	Established or not
30 days after primary vaccination						
DTcP-1 VS DTaP-IPV-Hib						
PT	1.16	(1.05,1.27)	0.67	Yes	1	Yes
FHA	0.93	(0.84,1.03)	0.67	Yes	1	No
DTcP-2 VS DTaP-IPV-Hib						
PT	1.29	(1.17,1.42)	0.67	Yes	1	Yes
FHA	1.29	(1.17,1.43)	0.67	Yes	1	Yes

Note: When the lower bound of two-sided the 97.5% CI on both sides of the point estimate ≥ 0.67, the non-inferiority is established. The superiority is established when the lower bound of the two-sided 97.5% CI is >1. When the non-inferiority test is established, the superiority test is performed.

The study evaluated immune responses by measuring antibody GMCs against DT, TT, PT, FHA, and PRN before (Day 0) and 30 days after primary vaccination (Day 30). At baseline, antibody levels were uniformly low across all groups (*P* > 0.05). By Day 30, all vaccines significantly boosted antibody levels, though differences emerged between groups. Compared to DTaP-IPV-Hib (GMC = 0.99), DT responses were similar in DTcP-1 (0.92, *P* = 0.065) and DTcP-2 (1.11, *P* = 0.051). For TT, DTaP-IPV-Hib (3.91) elicited higher responses than DTcP-1 (3.11, *P* < 0.001) but not DTcP-2 (4.26, *P* = 0.128). Pertussis-related antibodies showed more pronounced contrasts: PT levels were higher in DTcP-1 (75.63 vs. 65.35, *P* < 0.001) and DTcP-2 (84.23 vs. 65.35, *P* < 0.001) than in DTaP-IPV-Hib. Similarly, FHA responses in DTcP-2 (132.16) surpassed DTaP-IPV-Hib (102.13, *P* < 0.001), whereas DTcP-1 (94.98) showed no difference (*P* = 0.092). These results indicated that while all vaccines were immunogenic, DTcP-1 and DTcP-2 generally induced stronger pertussis-specific responses (PT) than DTaP-IPV-Hib, with DTcP-2 further excelling in FHA. TT responses favoured DTaP-IPV-Hib over DTcP-1 but matched DTcP-2, while DT responses were comparable across groups (Table 7).

**Table 7.** GMCs (IU/ml) of antibodies before vaccination and 30 days after primary vaccination.

	DTcP-1	DTaP-IPV-Hib	DTcP-2	P*	*P*#
Day 0					
DT (IU/ml)	0.00(3.35)	0.00(3.34)	0.00(3.42)	0.670	0.752
TT (IU/ml)	0.01(3.46)	0.01(3.79)	0.01(3.84)	0.685	0.505
PT (IU/ml)	0.64(3.91)	0.68(4.57)	0.83(4.23)	0.343	0.104
FHA (IU/ml)	2.68(2.92)	2.79(3.13)	3.05(3.16)	0.428	0.281
PRN† (IU/ml)	0.57(4.71)	0.63(4.61)	0.60(5.59)	0.432	0.784
Day 30					
DT (IU/ml)	0.92(1.99)	0.99(2.00)	1.11(2.09)	0.065	0.051
TT (IU/ml)	3.11(1.80)	3.91(1.74)	4.26(1.82)	<0.001	0.128
PT (IU/ml)	75.63(1.82)	65.35(1.81)	84.23(1.82)	<0.001	<0.001
FHA (IU/ml)	94.98(1.70)	102.13(1.99)	132.16(1.77)	0.092	<0.001
PRN† (IU/ml)	195.50(2.10)	2.01(1.48)	236.33(2.04)	<0.001	<0.001

* Comparison between DTaP-IPV-Hib and DTcP-1 groups.

# Comparison between DTaP-IPV-Hib and DTcP-2 groups.

† PRN antigen is a component of DTcP but is absent from the DTaP-IPV-Hib.

The numbers in parentheses were the SD value.

Table 8 showed the five antigens assessed 30 days after primary vaccination. It was indicated that the GMI for DT, TT, PT, and FHA were generally comparable between the DTaP-IPV-Hib, DTcP-1, and DTcP-2 groups, with no statistically significant differences observed in the pre-specified comparisons. Specifically, the GMIs for DT were 298.52, 295.51, and 327.69 for the DTaP-IPV-Hib, DTcP-1, and DTcP-2 groups, respectively. For TT, the GMIs were 692.06, 589.78, and 817.05, with *P*-values of 0.070 and 0.237 for the respective comparisons. Similarly, for PT (118.14, 95.90, 101.36; *P* = 0.055 and *P* = 0.332) and FHA (36.65, 35.45, 43.40; *P* = 0.784 and *P* = 0.059), the differences between groups did not reach statistical significance.

**Table 8.** GMIs of antibodies 30 days after primary vaccination.

	DTcP-1	DTaP-IPV-Hib	DTcP-2	P*	*P*#
Day 30					
DT	295.51(4.33)	298.52(4.50)	327.69(4.41)	0.759	0.648
TT	589.78(4.26)	692.06(5.12)	817.05(4.66)	0.070	0.237
PT	118.14(4.56)	95.90(5.49)	101.36(4.96)	0.055	0.332
FHA	35.45(3.41)	36.65(4.25)	43.40(3.82)	0.784	0.059
PRN†	344.86(6.34)	3.16(4.77)	390.66(7.10)	<0.001	<0.001

* Comparison between DTaP-IPV-Hib and DTcP-1 groups.

# Comparison between DTaP-IPV-Hib and DTcP-2 groups.

† PRN antigen is a component of DTcP but is absent from the DTaP-IPV-Hib.

The numbers in parentheses were the SD value.

Table 9 presented the seropositive rates of antibodies before and after primary vaccination. At baseline (Day 0), all groups showed low seropositivity rates with no significant differences. For DT, rates ranged from 15.42% to 19.37% across groups, while TT rates varied between 28.79% and 32.46%. Pertussis-related antigens demonstrated particularly low baseline seropositivity, with PRN showing the highest rates (6.43–12.04%) and PT the lowest (0.26–1.57%). No statistically significant differences were observed between DTaP-IPV-Hib and either DTcP-1 or DTcP-2 groups for any antigen at baseline (all *P* > 0.05). By Day 30 post-vaccination, dramatic increases in seropositivity were observed for all vaccines. Both DT and TT reached 100% seropositivity in all groups. For pertussis antigens, PT and FHA showed high seropositivity rates (>96% and >98% respectively) across all vaccines, with no significant differences between groups. These results demonstrate that all vaccines effectively induced immunity against DT and TT, DTcP-1 were also markedly in generating PT antibodies. The findings suggest important differences in the immunogenicity profiles of these vaccines, particularly regarding pertussis component responses.

**Table 9.** Seropositive rate of antibodies before vaccination and 30 days after primary vaccination.

	DTcP-1	DTaP-IPV-Hib	DTcP-2	P*	*P*#
Day 0					
DT	15.42% (11.98,19.40)	17.30% (13.81,21.25)	19.37% (15.53,23.70)	0.471	0.448
TT	28.79% (24.34,33.57)	31.75% (27.34,36.43)	32.46% (27.79,37.41)	0.359	0.830
PT	0.26% (0.01,1.42)	0.95% (0.26,2.41)	1.57% (0.58,3.39)	0.420	0.633
FHA	2.31% (1.06,4.35)	3.32% (1.83,5.50)	5.24% (3.23,7.97)	0.390	0.177
PRN†	6.43% (4.20,9.34)	9.48% (6.86,12.68)	12.04% (8.95,15.73)	0.110	0.240
Day 30					
DT	100.00% (99.06,100.00)	100.00% (99.13,100.00)	100.00% (99.04,100.00)	-	-
TT	100.00% (99.06,100.00)	100.00% (99.13,100.00)	100.00% (99.04,100.00)	-	-
PT	98.46% (96.67,99.43)	96.68% (94.50,98.17)	98.43% (96.61,99.42)	0.103	0.112
FHA	99.74% (98.58,99.99)	98.58% (96.93,99.48)	99.74% (98.55,99.99)	0.158	0.165
PRN†	99.49% (98.16,99.94)	1.90% (0.82,3.70)	100.00% (99.04,100.00)	<0.001	<0.001

* Comparison between DTaP-IPV-Hib and DTcP-1 groups.

# Comparison between DTaP-IPV-Hib and DTcP-2 groups.

† PRN antigen is a component of DTcP but is absent from the DTaP-IPV-Hib.

The numbers in parentheses were the 95% CI.

## Discussion

This phase III clinical trial evaluated the safety and immunogenicity of the novel DTcP vaccine in 2-month-old infants, comparing it to the licensed DTaP-IPV-Hib vaccine. The study also assessed the impact of different primary immunization schedules (2, 3, 4 months vs. 2, 4, 6 months) on immune responses. In this study, results demonstrated that the DTcP vaccine was well-tolerated and elicited robust immune responses, with notable differences in immunogenicity between the experimental and control groups, as well as between the two DTcP immunization schedules. The safety profiles of the DTcP and DTaP-IPV-Hib vaccines were comparable, with no vaccine-related serious adverse events reported in any group. This similarity in total adverse reactions incidence suggests that both DTcP and DTaP-IPV-Hib vaccines maintained an acceptable safety threshold. Local reactogenicity patterns revealed subtle but noteworthy variations. While erythema (2.44%–3.17%) and induration (0.30%–0.59%) showed comparable frequencies across groups (*P* > 0.05), swelling incidence was significantly higher in DTaP-IPV-Hib (4.83%) versus DTcP-2 (2.59%, *P* = 0.008). There was a difference in the incidence of fever among three groups, while the statistical difference was not shown significantly between DTcP-1 and DTaP-IPV-Hib (*P* = 0.056) or DTcP-2 and DTaP-IPV-Hib (*P* = 0.609). Besides, there was a markedly lower irritability frequency in DTcP-2 (0.07% vs 1.02%–1.40%, *P*  < 0.001). The transient nature of most reactions (resolution within 7 days) and absence of vaccine-related serious adverse events support the vaccines’ clinical suitability. The low incidences (<17% overall) and most are mild adverse reaction are particularly reassuring given established safety concerns with combination vaccines in young infants. The adverse reactions incidences in response to the DTcP vaccine administered at both 2, 3 and 4 months of age, and at 2, 4 and 6 months of age were comparable to those following vaccination with the DTaP-IPV-Hib control vaccine administered at 2, 3, and 4 months of age. These findings align with previous studies on acellular pertussis-containing vaccines, which have consistently shown favourable safety profiles in infants [14,15].

The immunogenicity analysis revealed that both DTcP and DTaP-IPV-Hib vaccines induced strong immune responses against diphtheria, tetanus, and pertussis antigens. Non-inferiority and superiority testing demonstrated that the DTcP-2 group elicited significantly higher GMCs for pertussis-related antibodies compared to DTaP-IPV-Hib. Specifically, anti-PT (point estimate = 1.29; 97.5% CI: 1.17-1.42) and anti-FHA (point estimate = 1.29; 97.5% CI: 1.17-1.43) responses met superiority criteria (lower CI bound ≥1). DTcP-1 (2, 3, 4-month schedule) showed non-inferiority for anti-PT and anti-FHA, and superiority for anti-PT (point estimate = 1.16; 97.5% CI: 1.05-1.27). Besides, the GMCs for PT, FHA, and PRN in the DTcP-2 group were 84.23, 132.16, and 236.33, respectively, compared to 65.35, 102.13, and 2.01 in the DTaP-IPV-Hib group. These results suggest that the DTcP vaccine, particularly when administered on a 2, 4, 6-month schedule, may provide superior protection against pertussis. In addition, it was found that extending the interval between two doses of the BNT162b2 mRNA vaccine (up to 44–46 weeks) not only did not raise new safety concerns but also induced stronger T-cell immunity and higher antibody levels [16]. In addition to high immune response induced by DTcP-2, the immunogenicity analysis comparing DTcP-1 and DTaP-IPV-Hib vaccines revealed significant differences in antibody responses 30 days post-vaccination. At baseline, GMCs for DT, TT, PT, FHA, and PRN were comparable between the two groups (all *P* > 0.05), indicating similar pre-vaccination antibody levels. However, by Day 30, DTcP-1 demonstrated significantly higher GMCs for PT (75.63 vs. 65.35, *P* < 0.001) compared to DTaP-IPV-Hib, suggesting a stronger immune response to the antigen. In contrast, DTaP-IPV-Hib elicited higher GMCs for TT (3.11 vs. 3.91, *P* < 0.001). The study demonstrated that PT can function as the primary virulence factor, with neutralizing antibodies preventing systemic toxicity and life-threatening complications [17]. Clinical findings support the significant as PT-specific antibodies are strongly correlated with protection against severe disease and mortality [18]. Besides, the robust anti-PRN response (236.33 IU/mL) induced by DTcP is particularly significant as epidemiological studies have established a direct correlation between circulating anti-PRN antibody levels and clinical protection. PRN functions as a critical virulence factor through its adhesive properties, with studies demonstrating that PRN-mediated protection occurs via blockade of Bordetella pertussis attachment to host cells [19]. The combination of strong PT neutralization (84.23 IU/mL) and comprehensive PRN-mediated protection positions DTcP as a potentially more effective vaccine against both disease severity and bacterial transmission compared to current acellular pertussis vaccines.

The marked difference in PRN response induced by DTcP was attributed to differences in vaccine formulations. During the production process, a non-animal-derived culture medium was developed for the production of the five antigenic components (FHA, PT, PRN, DT, and TT) included in the DTcP vaccine. Comprehensive studies and validation were conducted on the fermentation, purification, formaldehyde treatment/detoxification, and adsorption processes, ultimately establishing a stable production method. Additionally, studies indicated that the antigenic components and their concentrations in the pertussis (component) vaccine are well-defined, quality-controlled, and exhibit minimal batch-to-batch variation. For instance, Infanrix, a column chromatography-based purification process is employed to separately purify the pertussis antigens, which include PT, FHA, and PRN. The effective antigenic components are clearly identified and precisely quantified, ensuring consistent and controllable quality [20,21]. However, for control vaccine DTaP-IPV-Hib, which commercially named Pentaxim in China, was lack of PRN antigenic component, thereby cannot stimulate antibody against PRN in human body [22]. This study also has several limitations. First, this study was single-centre, which only conducted in Henan Province. The results may not be representative due to unique participants demographics, local medical practices and so on. Second, the assessment of vaccine efficacy was based on short-term immunological indicators, such as seroconversion rates and GMCs 30 days after primary vaccination. While, data on the durability of immune responses and the safety and immunogenicity results after the booster dose (18-24 months) have been collected and will be presented in subsequent publications. Third, larger population study should be considered to exclude the impact from geographic regions, different immunization background and so on. Post-marketing multi-centre surveillance in larger and more diverse populations will be conduct to get further safety and immunogenicity data.

In conclusion, the DTcP vaccine demonstrated a favourable safety profile and robust immunogenicity in 2-month-old infants, with both the 2,4,6-month and 2,3,4-month schedule groups showing strong immune responses, particularly for pertussis-related antigens. These findings support the potential use of DTcP as an option for early infant immunization, especially in high-burden regions. Building upon these results, we have further conducted a phase III head-to-head clinical trial comparing DTcP with both DTaP-IPV-Hib and co-purified DTaP vaccines in 3-month-old infants, the data from which will be presented in a forthcoming publication. Additional studies are warranted to evaluate the vaccine's long-term protection and real-world effectiveness against pertussis and other target diseases.

## Contributors

WZ, PW, TZ, HH, and YW conceptualized the study. XM, CW, and YW supervised the study. GF, FW, LW, JT, XW, XW, XS, WY, JG and LY contributed resources, curated data, and conducted the investigation. CW, LW, LY, and XM analysed and verified the data. WZ, CW, PW, GF, and FW wrote the original draft of the manuscript, which was revised by all authors. All authors had final responsibility for the decision to submit for publication.
